# Evaluating Probabilistic Traffic Load Effects on Large Bridges Using Long-Term Traffic Monitoring Data

**DOI:** 10.3390/s19225056

**Published:** 2019-11-19

**Authors:** Naiwei Lu, Yafei Ma, Yang Liu

**Affiliations:** 1 School of Civil Engineering, Changsha University of Science and Technology, Changsha 410114, China; lunaiweide@163.com (N.L.); liuyangbridge2@163.com (Y.L.); 2 School of Civil Engineering, Hunan University of Science, Zhuzhou 412007, China

**Keywords:** structural safety, traffic load, probabilistic extrapolation, bridge engineering, traffic load effect, stochastic traffic flow, level-crossing rate

## Abstract

With the steadily growing of global transportation market, the traffic load has increased dramatically over the past decades, which may develop into a risk source for existing bridges. The simultaneous presence of heavy trucks that are random in nature governs the serviceability limit for large bridges. This study investigated probabilistic traffic load effects on large bridges under actual heavy traffic load. Initially, critical stochastic traffic loading scenarios were simulated based on millions of traffic monitoring data in a highway bridge in China. A methodology of extrapolating maximum traffic load effects was presented based on the level-crossing theory. The effectiveness of the proposed method was demonstrated by probabilistic deflection investigation of a suspension bridge. Influence of traffic density variation and overloading control on the maximum deflection was investigated as recommendations for designers and managers. The numerical results show that the congested traffic mostly governs the critical traffic load effects on large bridges. Traffic growth results in higher maximum deformations and probabilities of failure of the bridge in its lifetime. Since the critical loading scenario contains multi-types of overloaded trucks, an effective overloading control measure has a remarkable influence on the lifetime maximum deflection. The stochastic traffic model and corresponding computational framework is expected to be developed to more types of bridges.

## 1. Introduction

With the steadily growing of the global transportation market, the highway traffic load has increased dramatically over the past decades. In 2018, the annual growth rate of freight traffic volume in China is roughly 6% that is three times that of European countries [1,2,3]. As a result, the current traffic load may exceed the design value in some design specifications that is evaluated based on traffic data a few decades ago. The traffic growth and truck overloading may result in risk sources for serviceability and safety of existing bridges. In fact, a large number of bridges all over the world has been damaged or even collapsed under heavy traffic loading [4,5]. In comparison with short-span bridges, a large bridge supports higher traffic loads including larger traffic volume and simultaneous presence of heavy trucks that govern critical state of large bridges [6,7,8]. This phenomenon leads to complications in evaluating traffic load effects on long-span bridges. Thus, the actual traffic patterns should be considered for probabilistic evaluation of traffic load effects on long-span bridges.

In general, structural health monitoring (SHM) systems are commonly used to investigate traffic load effects on bridges [9,10,11,12]. These monitoring data can be directly utilized to evaluate various conditions of the bridge, e.g., fatigue damage, serviceability, and durability [13,14,15,16,17,18]. In addition to monitoring bridge responses (stresses, displacements, and accelerations) with SHM systems installed in long-span bridges, an alternative approach is the numerical simulation with site-specific weigh-in-motion (WIM) measurements. A WIM system is usually installed with loop and piezo sensors under road pavement to monitor traffic loads dynamically without traffic interruption [19]. With the developments in sensor technology and computational methods [20], the WIM system has been developed for a REMOVE strategy in Europe [21]. In addition to traffic management, it can also provide a great amount of real traffic data for truck overloading identification and control. In practice, the WIM data has a wide range of applications in bridge engineering, such as live-load calibration in design specifications, fatigue reliability assessment, as well as lifetime maximum traffic load effects evaluation [22,23,24,25,26]. One of these achievements is the application of static load effects in the extrapolation of extreme value by conventional methods, such as general extreme value theory and level-crossing theory [27].

In addition to short and medium span bridges [28,29], long-span bridges have been investigated accounting for traffic loading patterns. The simultaneous presence of heavy trucks with mixtures of various statistical distributions makes the traffic pattern on long-span bridges more complicated [30]. The Monte-Carlo simulation (MCS) and cellular automaton (CA) technology are popular in this regard [31]. It has been demonstrated by researchers [32,33] that congested traffic flows have a significant influence on the maximum traffic load effect on long-span bridges. In this regard, Xia and Ni (2016) [34] investigated the extreme stress of Tsing Ma Bridge with a return period of 120 years using SHM data. Ruan et al. (2017) [35] presented a site-specific traffic load model for cable-stayed bridge, which can provide a reference for similar bridge. Lu et al. (2018) [36] investigated lifetime deflection of a cable-stayed bridge considering multiple traffic densities based on WIM data. Micu et al. (2019) [37] found maximum traffic load effect on a suspension bridge based on vehicle length from image data. Yu et al. (2019) [38] predicted the maximum of bridge load effects considering traffic growth using a non-stationary Bayesian method. Although extensive studies have been carried out to evaluate the lifetime maximum load effects with WIM measurements, the application of these data for assessing the serviceability of long-span bridges is essential. In addition, most of the studies in this field have focused on the extrapolation of short-span bridges. Due to the special traffic pattern (the traffic density and traffic gap) on long-span bridges, statistical extrapolation is still a challenge. One of the difficulties in this area is how to simulate the stochastic traffic flows on long-span bridges. Moreover, the probabilistic modelling of load effects requires time-consuming computations.

This study aims to evaluate probabilistic traffic load effects on long-span bridges using traffic monitoring data. Initially, millions of WIM measurements collected from a suspension bridge in China were introduced and utilized for probability density fitting of traffic parameters. A hybrid traffic simulation method was presented by combining MCS and CA approaches to simulate multiscale critical stochastic traffic loading scenarios. A methodology for evaluating probabilistic maximum traffic load effects was developed based on level-crossing theory. Case study of the lifetime deflection of a suspension bridge was conducted to demonstrate the effective of the presented traffic load model and corresponding computational framework. Daily maximum traffic load effects on were estimated via the critical influence lines of the bridge. The probabilistic extrapolation was conducted based on Rice’s level-crossing formula. Traffic growth and truck overloading control were considered in the parametric study to emphasize the application prospect of the present study.

## 2. Traffic Monitoring Data and Traffic Flow Simulation

### 2.1. Traffic Data from WIM System

**Figure 1:**
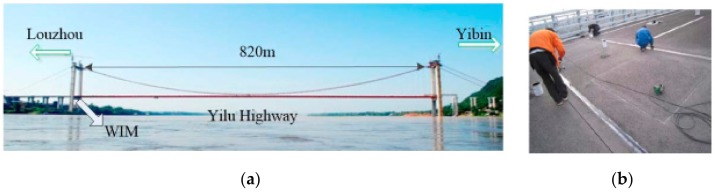
Onsite photos: (**a**) Nanxi Yangtze River Bridge; (**b**) weigh-in-motion (WIM) system during construction.

The traffic data in the present study were collected from a WIM system on a highway bridge crossing Yangtze River located at Yilu Highway in Sichuan, China. The traffic data were utilized as probabilistic database to simulate stochastic traffic flows. Some of the onsite photos are shown in Figure 1. A detailed illustration of these data can be found in [39,40], where these data have been used for fatigue reliability evaluation of orthotropic steel decks of the bridge. Table 1 summarizes the general information of these data. The maximum gross vehicle weights (GVWs) of individual truck were evaluated from the annual traffic data. The overloaded trucks were filtrated according to the Limits of Dimensions, Axle load, and Masses for road vehicle in the National Standard of the People’s Republic of China (GB 1589-2016) [41], where the threshold weights for 5-axle and 6-axle trucks are 500 and 550 kN, respectively.

It is well known that the driving speed impacts the WIM sensor data due to the dynamic effect. In the present study, the vehicle data collected from the WIM system has been comprehensively processed in the packed bundled software. In another word, the WIM system provided the vehicle weight data directly with consideration of driving effects and environmental effects. Since the vehicle types are various, it is difficult to consider all types of vehicles in actual configurations. This study divided the vehicle into six types according to the number of axles. Therefore, a type of vehicle includes multi-configurations, and the representative configuration with highest probability density was adopted in the present study. A detailed illustration of the vehicle classification utilized in the present study is shown in Table 2.

**Figure 2:**
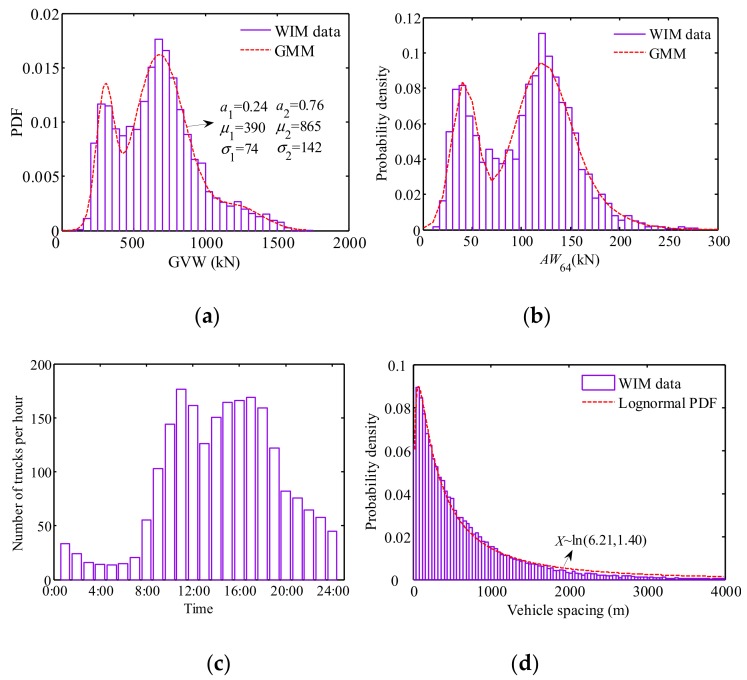
Probability densities of traffic parameters based on WIM data: (**a**) gross vehicle weight (GVW) of 6-axle trucks; (**b**) the 4th axle weight of the 6-axle trucks; (**c**) traffic density; (**d**) vehicle spacing in the slow lane.

For the concentration of the current study, the V6 vehicles were taken as an example to demonstrate the probability distribution of vehicle weights. The probability models for GVWs and axle weights are shown in Figure 2a,b, respectively. It is observed that the vehicle weight follows a bi-modal Gaussian distribution, which is well fitted by a Gaussian mixture model (GMM). The two peaks of the histograms correspond to the empty state and fully loaded state of trucks. With the growth of truck loading, the spacing between the two peaks will become wider. The bi-modal Gaussian distribution behavior of the truck loads are in accordance with most of literatures [24]. In addition to the GMM, the alternative model such as Lognormal mixture models and Gumbel mixture models can be also used for the fitting.

Compared to shot or medium span bridges, the vehicle spacing/gap has a remarkable influence on the load effect on longer-span bridges. The vehicle spacing in the present study was calculated based on the driving speed and duration of two following vehicles. Figure 2c,d plots the statistical histogram of traffic volume and vehicle spacing in the slow lane, respectively. It is observed that busy traffic is mostly concentrated between 9:00 and 19:00, which is in agreement with human normal working time. Since this study focuses on the serviceability limit state of bridges, it is reasonable to select the busy traffic for the following vehicle spacing statistical analysis.

Note that these PDFs shown in Figure 2 were fitted using an annual traffic data including weekdays and weekends. In practice, the traffic patterns on weekdays and weekends are different. Some researchers excluded the traffic data on weekends. For instance, OBrien et al. [29] considered 250 working days per year to investigate the maximum traffic load effect. However, in the present study all of the traffic data were included to make the fitted PDFs more comprehensive.

### 2.2. Traffic Flow Simulation

With the traffic probability model, it is important to utilize a reasonable approach to simulate the actual traffic pattern. In general, the conventional MCS is a popular approach for simulating traffic flows [42]. However, MCS is not an effective or efficient tool for simulating the acceleration and deceleration of an individual vehicle. In order to address these shortcomings, numerous traffic models have been developed, where an extensive review has been conducted by Pel et al. (2012) [43]. The CA model was developed by numerous researches to overcome the MCS shortcomings in driving behavior of individual vehicles in local scale. More details regarding the application of CA model in traffic simulation can be found in Chen et al. (2013) [44]. In the present study, the CA model is used to simulate the local driving behavior of individual vehicles on the bridge.

In a CA model, the traffic space is assumed as a large number of cell grids, and then each vehicle is placed in a cell. At each time step, each vehicle accelerates, decelerates, or moves depending on the predefined rules shown in Equation (1) to Equation (3) [45].

(1) $$\mathrm{If}\;v_{t}^{i}<v_{\max}\;\mathrm{and}\;{\mathrm{gap}}_{t+1}^{i}\geq v_{t}^{i}+1,\;\mathrm{then},\;v_{t+1}^{i}=v_{t}^{i}+1$$

(2) $$\mathrm{If}\;{\mathrm{gap}}_{t+1}^{i}\leq v_{t}^{i}+1,\;\mathrm{then},\;v_{t+1}^{i}=v_{t}^{i}-1$$

(3) $$\mathrm{Otherwise},\;v_{t+1}^{i}=v_{t}^{i}$$

where, $v_{t}^{i}$ and $v_{t+1}^{i}$ denote the velocity in terms of cell/s for the *i*th vehicle at time *t* and *t*+1, respectively; *v*_max_ denotes the speed limit, and ${\mathrm{gap}}_{t+1}^{i}$ is the gap between two vehicles in a traffic lane in terms of cell/s.

**Figure 3:**
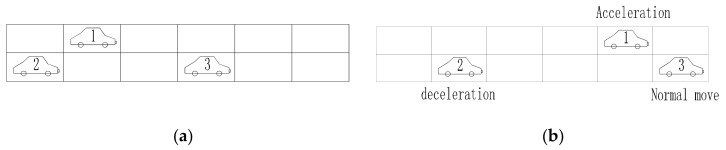
Cellular automaton (CA) rules: (**a**) *T* = *t*; (**b**)*T* = *t* + 1.

**Figure 4:**
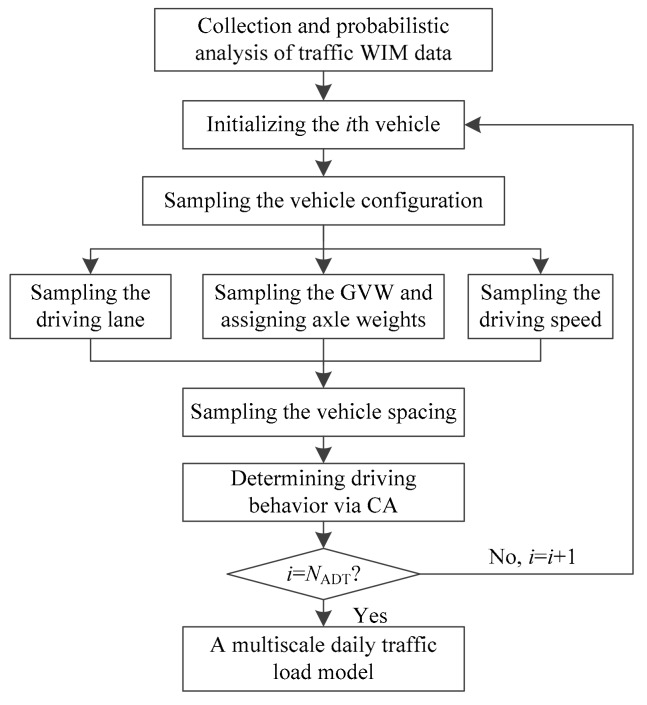
A hybrid traffic simulation method based on Monte Carlo simulation (MCS) and CA.

In order to capture the multi-scale behavior of traffic loading on bridges, MCS and CA were combined to simulate a hybrid model of stochastic traffic load. The global-scale vehicles are simulated based on actual probability distributions as shown in Figure 2 via MCS, and the local-scale driving behavior of an individual vehicle is controlled by CA rules as shown in Figure 3. A general framework of the hybrid method is summarized in Figure 4.

**Figure 5:**
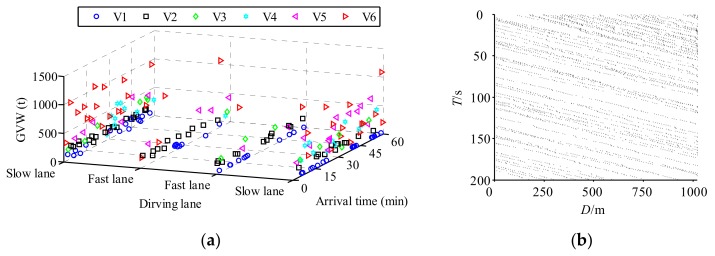
Traffic flow simulations: (**a**) Global simulation via MCS; (**b**) local dynamic behavior of individual trucks via CA.

In Figure 4, *N*_ADT_ is the average daily traffic volume. The detailed steps are shown as follows. Initially, the collected WIM data was filtrated to remove the invalid vehicle data, where the probabilistic traffic parameters, such as the vehicle type proportion, and GVW were evaluated. Secondly, the global parameters of individual vehicles including vehicle configurations, driving lane, driving speed, and GVWs were generated via MCS. Thirdly, the vehicle spacing/gap considering dense traffic or free flowing traffic was simulated. Finally, the individual vehicle driving behavior including deceleration and changing driving lanes was determined via CA rules. Based on the above procedures, a multi-scale traffic model can be simulated, where the global-scale parameters were considered in MCS, and the local-scale behavior is considered in CA rules. Based on the statistical database of the WIM measurements presented above, a multiscale stochastic traffic model was simulated as shown in Figure 5.

Figure 5a shows the large scale traffic in 60 min simulated via MCS, where each marker indicates a vehicle including vehicle types, driving lanes, and GVWs. It is observed that the slow lane includes numerous overloaded trucks, especially for V5 and V6 trucks. Figure 5b shows the dynamic driving behavior of the vehicles in 200 s in local scale coordination via CA rules. In the CA rule, the length of each cell is 5 m, the time step is 1 s, the number of cells is 200, the density of vehicles is 0.027, and the probability of changing driving lanes is 0.5. The pattern of changing driving lane is similar to overtaking. This means if a vehicle driving following another vehicle, the vehicle behind has a 50% probability to change the lane and overtake the front vehicle, if the adjacent lane is free. It has also 50% probability to following the front vehicle. This value and driving pattern are referred to Chen and Wu [45].

### 2.3. Critical Loading Scenarios

Since larger bridges usually have longer influence lines, the traffic loading pattern will impact the load effect significantly. However, it is a time-consuming process to compute numerous daily traffic load effects. In order to save the computational effort, this study searched the daily critical loading scenario which governs the maximum traffic load effect. A critical loading scenario approach corresponds to daily maxima, and thus it is needless to use the entire daily traffic data to estimate the daily load effect. However, it is reasonable to select the correct critical loading scenario from the daily traffic flow.

**Figure 6:**
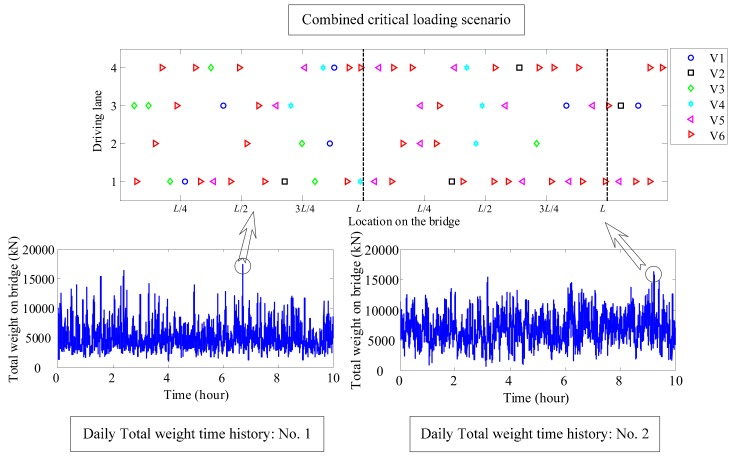
An example of identifying daily critical traffic loading scenarios.

Based on the above assumption, the following steps were used to identify the critical traffic loading scenarios. Initially, the loading range was determined as the bridge length. Secondly, the traffic flow moves forward to evaluate the time-dependent total weight of the vehicles on the bridge. Subsequently, identify the maximum total weight and the corresponding loading scenarios. Finally, these identified critical loading scenarios were combined to generate a simplified multi-day traffic flow model. An illustrative example for generating the critical loading scenario is shown in Figure 6.

In Figure 6 the bottom figures show two histories of the total weight on the bridge in 10 h computed using the stochastic traffic model. Daily maximum total weight on the bridge were found in the histories, and the corresponding loading scenarios were identified in the daily traffic model as shown on the top of Figure 6. Finally, the identified critical loading scenarios were combined together to generate a combined critical loading scenario, that can be used for the following traffic load evaluation. It is worth to note that critical loading approach identifies approximately 0.1% of the daily traffic data to evaluate the extreme load effect, which has greatly reduced the time-consuming computation.

Once the critical scenarios are completely identified, these loading scenarios can be combined with enough spacing to generate a combined critical loading scenario as shown in Figure 6. With the bridge influence lines, the load effect histories can be evaluated. Subsequently, the level-crossing rate will be fitted, which will be used for extrapolation.

## 3. Methodology for Extrapolating Maximum Traffic Load Effects

### 3.1. Theoretical Basis

**Figure 7:**
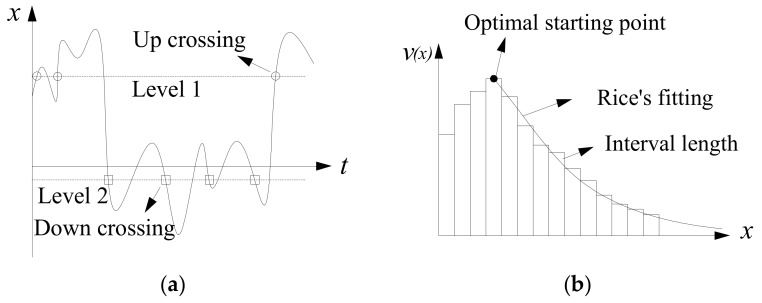
Rice’s level-crossing principle: (**a**) counting number of crossings; (**b**) fitting level-crossing rate.

Rice’s level-crossing principle [46], as shown in Figure 7, was utilized in the present as a probabilistic basis for extrapolation. Note that Rice’s formula for extrapolation is only effective for random variables following Gaussian distribution [47]. In general, the traffic load effect can be assumed following Gaussian distribution according to the opening literatures [2,30]. Thus, the Rice’s formula can be used in the present study for extrapolating traffic load effects.

As shown in Figure 7, the up-crossing rate is the kernel parameter to extrapolate maximum value. It is common to use *v*(*x*) for a reference period and a threshold value [48]:

(4) $$v(x)=\frac{\sigma^{\prime }}{2\pi \sigma }\exp\left[-\frac{{\left(x-m\right)}^{2}}{2\sigma_{}^{2}}\right]$$

where *x* is the load effect which is random in nature, *m* and *σ* are the mean value and standard deviation, respectively. When using Rice’s formula for extrapolating, a cumulative distribution function (CDF) is essential which is given by

(5) $$F(x)=\exp\left[-v_{0}Rt\exp\left(-\frac{1}{2}{\left(\frac{x-m}{\sigma }\right)}^{2}\right)\right]$$

where *v*_0_ is equal to *σ*’/2π, and *R_t_* is the return period.

It is worth noting that the extrapolating accuracy mostly depends on the starting point and the standard derivation. Therefore, it is important to select the optimal value to start the fitting with:

(6) $$x_{\max}(R_{t})=m_{\mathrm{opt}}+\sigma_{\mathrm{opt}}\sqrt{2\ln(v_{0}{}_{,\mathrm{opt}}R_{t})}$$

where, *m*_opt_, *σ*_opt_ and *v*_0,opt_ are the optimal mean value, the optimal standard deviation, and the optimal original level-crossing rate, respectively. The optimal fitting parameters can be evaluated from the Kolmogorov test with a conventional confidence level between 0.9 and 1. Detailed optimal procedure can be found in Cremona [48], where the application on extrapolating traffic loads and load effects on multi-span bridges were demonstrated.

Based on Rice formula for extrapolation, the first-passage probability of the random process during a period under a threshold can be evaluated by:

(7) $$p(a,\tau )≅1-\exp\left[-\int_{0}^{\tau }v(a,t)dt\right]$$

where, *a* is a threshold of random variable, and *t* is the reference period of the bridge, which is a bridge lifetime. The above formulations provide a reasonable approach to estimate maximum load effects on long-span bridges. With the consideration of these load effects, probabilistic modelling can then be carried out by the Rice formula.

### 3.2. Computational Framework

**Figure 8:**
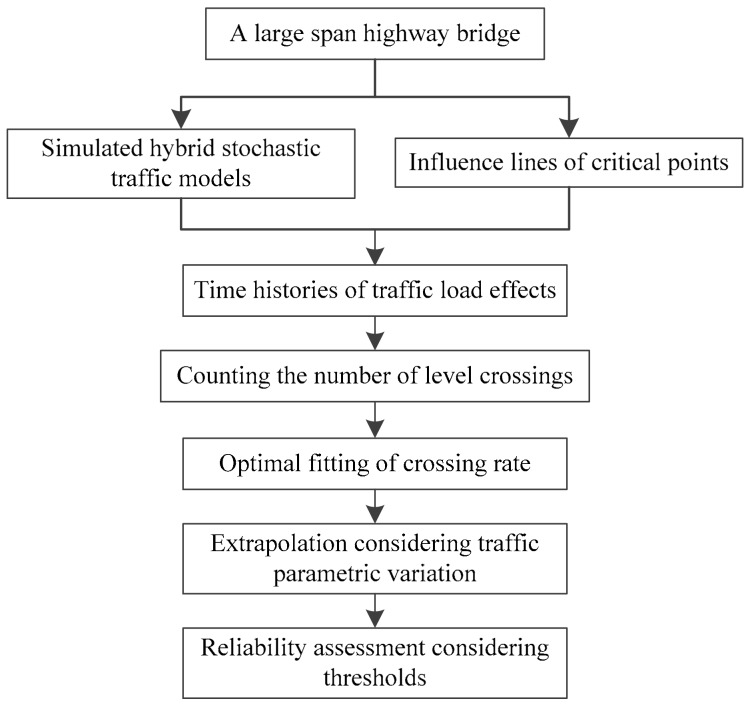
Computational framework for probabilistic modelling and assessment of traffic load effects.

Based on the theoretical basis illustrated above, a general computational framework is presented to connect the hybrid traffic model and the probabilistic the load effect analysis. The main procedures are summarized in Figure 8.

The main procedures of the computational framework in Figure 8 can be simplified into three steps. Firstly, simulate the traffic load effect histories of the bridge under simulated traffic load model, where the traffic probability and driving behavior are included. Secondly, fit the optimal level-crossing model using the simulated histories. The fitting accuracy step has a significant influence on the extrapolation result. Finally, evaluate the maximum value considering a return period, and the reliability of the bridge can also be evaluated with a component resistance or threshold.

## 4. Case Study

### 4.1. Prototype Suspension Bridge

**Figure 9:**
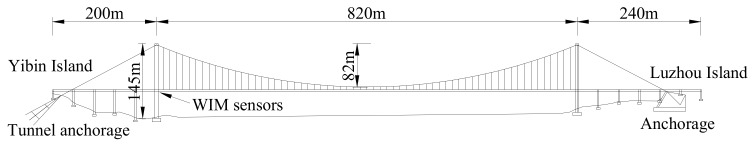
Dimensions of the suspension bridge.

The Nanxi Yangtze River Bridge is a suspension bridge with a main span of 820 m at Yilu Highway located in Sichuan, China. This bridge is chosen herein as a prototype bridge to evaluate the maximum deflection using traffic monitoring data. The WIM measurements mentioned above were monitored for this bridge. The dimensions of the elevation layout are shown in Figure 9. Elaborate information of the bridge is given by Liu et al., (2015) [49]. Table 1 indicates that a great number of overloaded trucks pass over the bridge every day. Therefore, it is an urgent task to evaluate the serviceability of the bridge under the current and future traffic loads.

### 4.2. Probabilistic Modelling of the Extreme Load Effects

**Figure 10:**
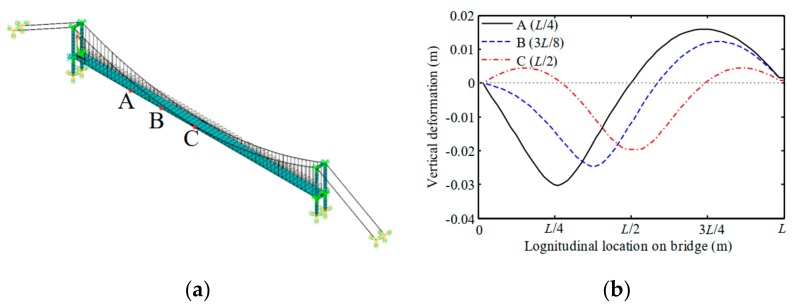
Critical deformation influence lines of the suspension bridge: (**a**) Critical locations; (**b**) influence lines.

For the serviceability assessment of the bridge under traffic load, the girder deflection is selected for the probabilistic modeling extreme load effects. The framework shown in Figure 8 is utilized as an outline for the computation. Initially, a finite-element model was built to obtain the influence lines under moving vehicle load. The geometric dimensions, material properties, and initial cable forces were determined by the design parameters. The influence lines of the bridge under a moving load of 100 kN were estimated in the bridge finite element model. Figure 10 plots the deformation influence lines of the *L*/4, 3*L*/8, and *L*/2 points of the bridge girders. It is observed that the quarter-span is the most critical point for the maximum deflection of girders. This is in correspondence with the vertical mode shape of the bridge girders which is asymmetric. Liu et al., (2015) [49] presented the similar influence lines using the deflection monitoring data from a connected pipe system of a suspension bridge. Therefore, this study focuses on the deflection of the *L*/4 point.

**Figure 11:**
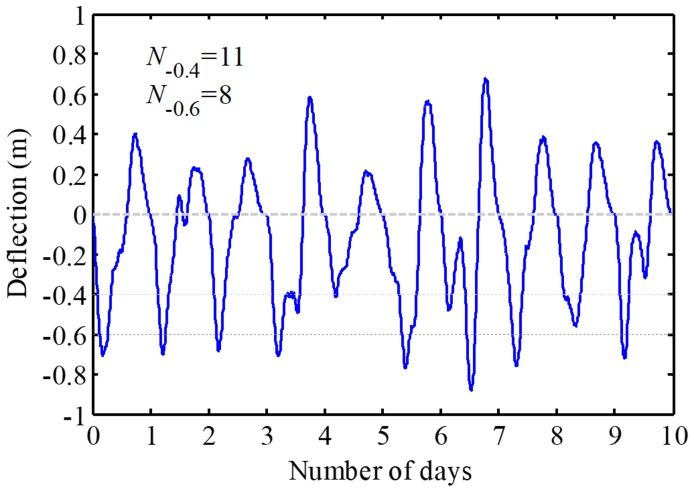
Time-history samples of the bridge deformation under identified critical traffic loads.

**Figure 12:**
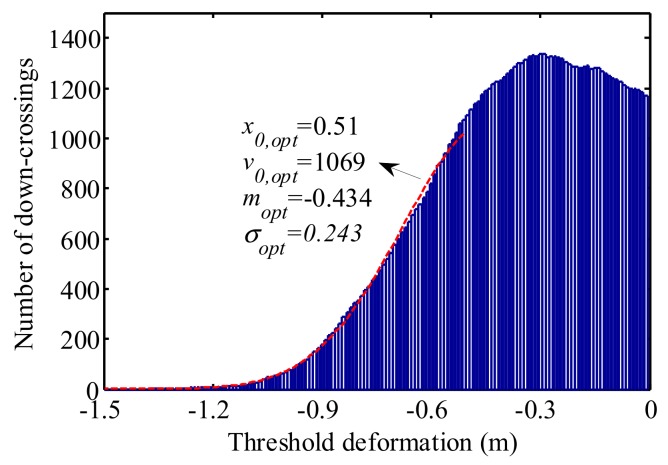
Histograms of Rice’s level-crossing rate.

Deflection-time histories of the bridge were evaluated considering critical loading scenarios given in Figure 6. The deflection-time history of the *L*/4 point under 10-day critical loading scenarios is shown in Figure 11, where *N*_−0.4_ and *N*_−0.6_ are the numbers of down-crossings of the threshold deflections of −0.4 m and −0.6 m, respectively. It is obvious that the number of crossings decreases with the increase of the threshold deflection. Based on 1000-day traffic loading scenarios, the histograms and fitted optimal curves were estimated as shown in Figure 12.

In Figure 12, the optimal starting point *x*_0,opt_ = 0.51 m, the optimal number of down-crossings *v_0,opt_* = 1069, the optimal mean value *m*_opt_ = −0.434 m, the optimal standard deviation *σ_opt_* = 0.243 m and the optimal length of the intervals is 0.01 m. It is observed that Rice’s fitting is close to the tail of the histograms. This indicates that the fitted curve has a good quality of extrapolation. Subsequently, 1000-day block extreme values were utilized to investigate the deviation between GEV results and Rice’s fitting.

**Figure 13:**
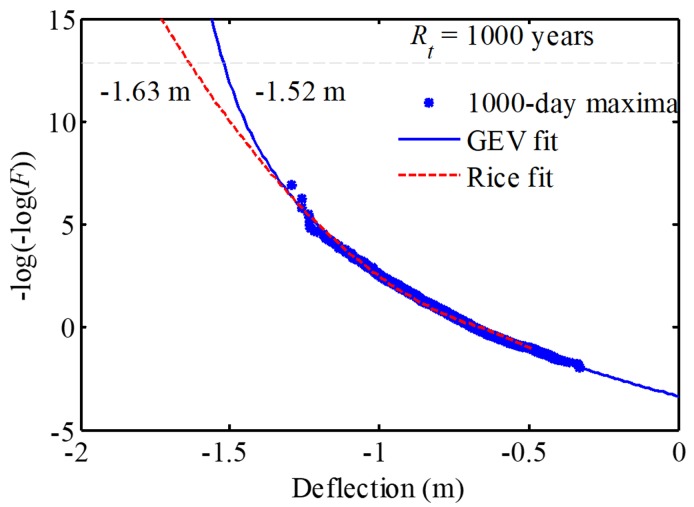
Comparison of Rice and generalized extreme value (GEV) fittings plotted on Gumbel probability paper.

Figure 13 plots the GEV fitting and the Rice’s fitting based on 1000 maxima. It is observed that both fittings are close to the original data, but they have a different trend for the extrapolations. The extrapolated maximum deflections in the 1000-year return period are −1.63 m and −1.52 m, respectively. The deviation can be clarified in a future work by utilizing advanced fitting approaches and more data. According to the General Code for Design of Highway Bridges and Culverts (D60-2015) in China [50], threshold value should be less than the threshold *a* = *L*/400 = 2.04 m for the prototype bridge. Therefore, the current traffic load is far away for the serviceability limit state of the bridge.

### 4.3. Parametric Study

**Figure 14:**
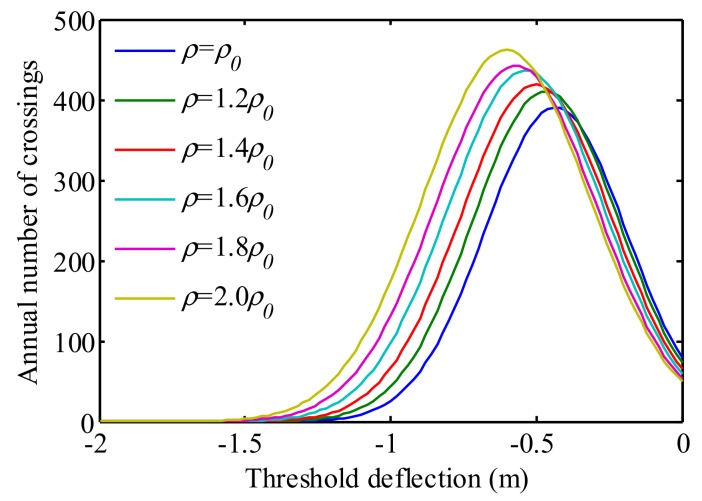
Influence of traffic density on the level-crossing rate.

The above investigation was conducted without considering traffic growth during the bridge lifetime. In practice, the traffic volume has a relationship with the traffic density [51]. Thus, in order to investigate the influence of traffic growth on the traffic load effect, this study supposes that the future traffic densities are 1.2, 1.4, 1.6, 1.8, and 2.0 *ρ*_0_, respectively. The estimated down-crossing rates are shown in Figure 14. It is obvious that the traffic density growth results in the increase of *v*_0_, *m*, and *σ* in the fitting.

As observed from Figure 14, with the increase of traffic density the number of crossings increases and moves to left. This phenomenon can be explained by the fact that the traffic density increase leads to the decrease of the vehicle gap on the bridge, and thus leads to the increase in the loading density. Therefore, the higher deflection value has a larger number of crossings. It is also observed that the peak of the curve moves to left, which is caused by the increase of the means total weight on the bridge.

**Figure 15:**
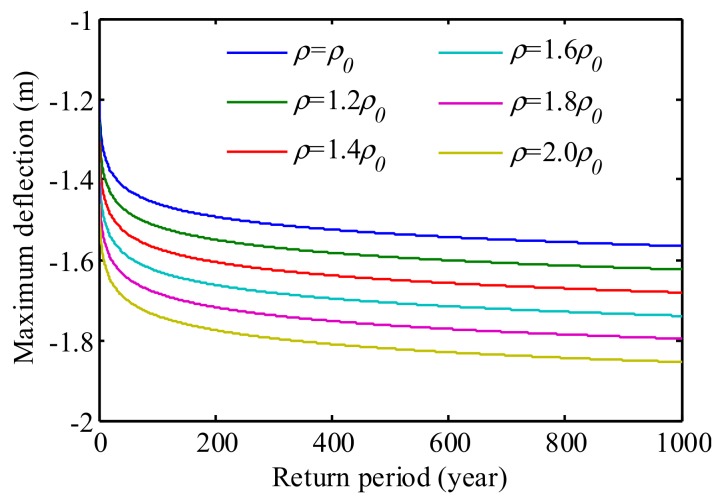
Maximum deflections accounting for growing traffic loads.

By utilizing such probabilistic model, both the maximum deflection and the probability of exceedance of the threshold deflection can be estimated via Equations (6) and (7). Subsequently, the following studies focus on parametric studies. With the level-crossing model illustrated above, the maximum deflections and the probability of exceedance in bridge lifetime was evaluated. Initially, the maximum deflections in a 1000-year return period were estimated as shown in Figure 15. It is observed that when the traffic density doubles, the bridge maximum deflection in 1000-year return period increases from −1.58 to −1.85 m. In addition, the extrapolation of the deflection increases linearly with the linear growth of the traffic density.

**Figure 16:**
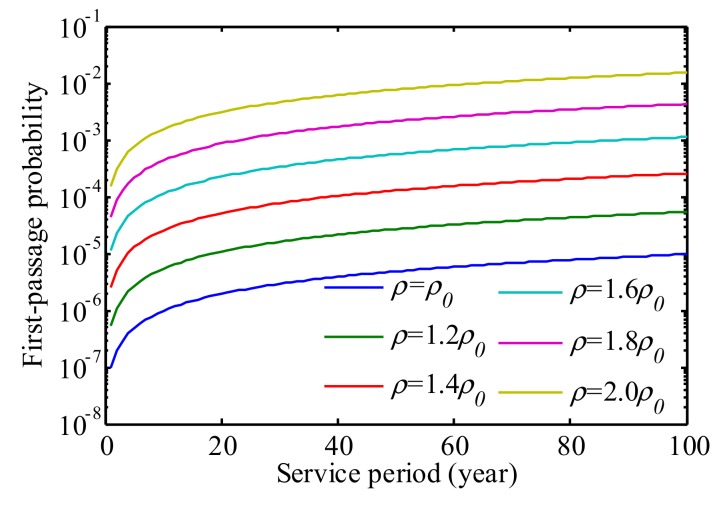
First-passage probability accounting for traffic growth.

The first-passage probabilities of the bridge under the predefined threshold were estimated accounting for traffic growth based on Equation (7). Figure 16 plots the probabilities of failure of the bridge in its lifetime. It is observed that when the traffic density is doubled, the first-passage probability in the bridge lifetime increases from 9.7 × 10^−6^ to 1.5 × 10^−2^. In addition, the increasing rate of the first-passage probability slows down.

**Figure 17:**
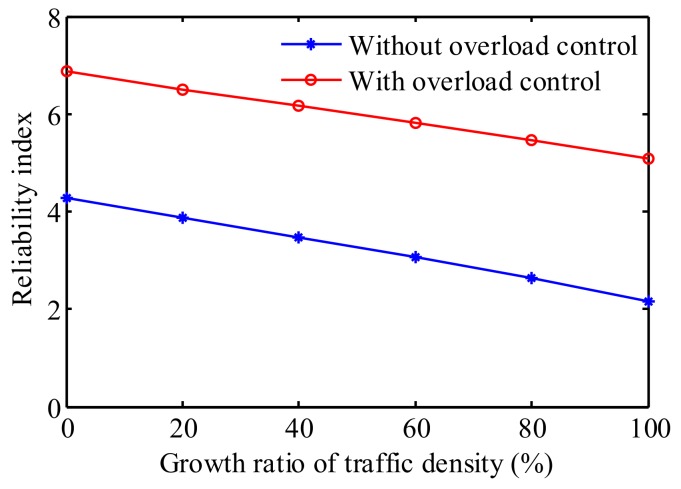
Reliability indices of the deformation first-passage of the bridge accounting for traffic growth and truck overloading control.

Since the extreme values in the time-history are mostly associated with overloaded trucks, the truck overloading control deserves investigation. In China, the threshold legal weight for 6-, 5-, 4-, 3-, and 2-axle trucks are 55, 40, 30, and 20 t, respectively. The stochastic traffic load model was updated, and the reliability indices of the serviceability of the bridge accounting for both traffic growth and truck overloading control are shown in Figure 17.

As observed from Figure 17, without consideration of truck overloading control, the reliability indices are 4.27 and 2.16 for the current and future traffic condition, respectively. However, the reliability index shows a remarkable increase under the overload control. The corresponding reliability indices for the current and future traffic are 6.87 and 5.08, respectively. The effective increase of the reliability index indicates that the truck overloading control is essential for bridges with heavy traffic, especially under the condition of growing traffic load.

## 5. Conclusions

This study investigated probabilistic traffic load effects on large bridges based on traffic monitoring data. The traffic load effects were simulated based on long-term monitored highway traffic data via a novel multi-scale traffic model. It has been demonstrated that the proposed traffic simulation method has the capacity of capturing the traffic probability parameters in global scale and the individual truck driving behavior in local scale. A general computational framework was presented for maximum traffic load effect simulation. The simulated traffic model and presented computational framework were applied to lifetime deflection evaluation of the main girder of a suspension bridge. The parametric study indicates that the traffic volume growth has a higher influence on the maximum deformation of the bridge, and thus leads to a higher probability of failure in bridge lifetime. In addition, the truck overloading control is a very efficient way of ensuring the bridge reliability, and thus the truck overloading control is essential for heavy traffic area. In the view of practical application, the numerical results can provide a theoretical basis for truck overloading control.

In addition to evaluating the maximum deformation of large bridges, the proposed traffic model and computational framework can be utilized for more aspects, such as traffic induced fatigue stresses, girder acceleration, and cable forces. However, further studies are necessary to improve the computational efficiency and accuracy. Probabilistic machine learning approaches can be utilized as a surrogate model to replace the time-consuming finite element runs. Subsequently, a reasonable traffic growth model will be considered in the future study. Finally, the parametric studies on starting points of the fitted level-crossing model should be conducted and compared with the GEV results.

## Figures and Tables

**Table 1:** Introduction of the WIM measurements.

Items	Values
Duration	Jan. 1, 2017 to Dec. 31, 2017
Recording days	365
Daily truck traffic volume	982
Traffic lanes	4
Maximum GVW (kN)	1, 524
Total overloaded trucks	12521

**Table 2:** Vehicle configurations and proportions.

Vehicle Type	Description	Configuration (m)	Proportion (%)
V_1_	Light truck	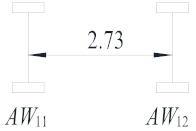	27.59
V_2_	2-axle truck	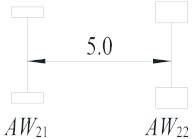	31.23
V_3_	3-axle truck	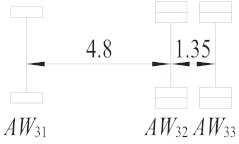	4.15
V_4_	4-axle truck	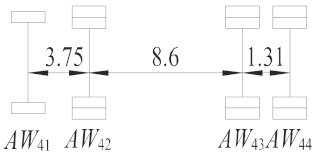	10.44
V_5_	5-axle trucks	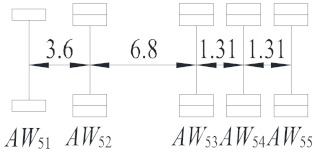	10.78
V_6_	6-axle truck	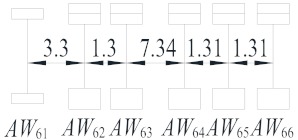	15.82
